# Ovicidal effect of eucalyptus wood vinegar on gastrointestinal nematodes’ eggs from sheep

**DOI:** 10.14202/vetworld.2025.1156-1167

**Published:** 2025-05-13

**Authors:** Yandra Thais Rocha da Mota, Alexandre Santos Pimenta, Moacir Franco de Oliveira, Ana Karolinne de Alencar França, Andressa Marcelly Silvestre Pereira, Rafael Rodolfo de Melo, Thays Vieira da Costa Monteiro, Maíra Fasciotti, Lúcio César Dantas de Medeiros, Ana Carla Diógenes Suassuna Bezerra

**Affiliations:** 1 Diagnostic and Experimental Parasitology Laboratory, Graduate Program in Development and Environment – PRODEMA, Universidade Federal Rural do Semi-Árido – UFERSA, Mossoró City, Rio Grande do Norte State, Brazil; 2 Graduate Program in Forest Sciences – PPGCFL, Universidade Federal do Rio Grande do Norte – UFRN, Macaíba City, Rio Grande do Norte State, Brazil; 3 Department of Animal Sciences, Universidade Federal Rural do Semi-Árido – UFERSA, Mossoró City, Rio Grande do Norte State, Brazil; 4 Laboratory of Wood Technology, Department of Agronomy and Forest Engineering, Universidade Federal Rural do Semi-Árido – UFERSA, Mossoró, Rio Grande do Norte State, Brazil; 5 Laboratory of Organic Analysis, Instituto Nacional de Metrologia, Qualidade e Tecnologia, Av. Nossa Sra. das Graças, 50, Xerém, Duque de Caxias 25250-020, Rio de Janeiro, Brazil

**Keywords:** anthelmintic resistance, egg hatch test, eucalyptus vinegar, *Origanum majorana*, phenolic compounds, sustainable parasite control

## Abstract

**Background and Aim:**

Gastrointestinal nematodes (GINs) significantly impair small ruminant production globally, particularly in tropical regions. Anthelmintic resistance due to the indiscriminate use of synthetic drugs has necessitated the search for sustainable, plant-based alternatives. Eucalyptus wood vinegar (WV), a by-product of biomass pyrolysis, possesses bioactive compounds with potential anthelmintic activity. This study aimed to assess the *in vitro* ovicidal efficacy of eucalyptus WV and WV derived from co-pyrolysis of eucalyptus wood with *Origanum majorana* (marjoram) against eggs of GINs from naturally infected sheep.

**Materials and Methods:**

WV samples were produced through controlled pyrolysis and refined through sequential vacuum distillation. Egg hatchability tests were performed using five WV concentrations (0.3125%–5% g/100 mL), with thiabendazole as a positive control and distilled water as a negative control. Egg counts, species identification, and scanning electron microscopy (SEM) were conducted to evaluate structural changes. The chemical compositions of the WVs were characterized using gas chromatography-mass spectrometry (GC/MS).

**Results:**

Both WVs exhibited significant ovicidal activity, with eucalyptus WV achieving 97% inhibition at 1.25%, and the marjoram-enriched WV reaching 100% inhibition at 5%. GC/MS analysis revealed the presence of phenolic compounds, furfural, thymol, and eucalyptol, the latter two being exclusive to the marjoram formulation. SEM micrographs confirmed morphological deformations in treated eggs, including loss of symmetry and membrane integrity. The synergistic interaction among bioactive components, particularly thymol, eucalyptol, and furfural, is proposed as the mechanism enhancing ovicidal activity.

**Conclusion:**

Eucalyptus WV, particularly when enriched with *O. majorana* through co-pyrolysis, exhibits potent ovicidal effects against GINs in sheep. These findings support the potential use of WVs as eco-friendly anthelmintic alternatives in integrated parasite management strategies for small ruminants.

## INTRODUCTION

According to the Brazilian Institute of Geography and Statistics [1], the national sheep population in Brazil was estimated at 18 million in 2021, with the Northeast region accounting for approximately 14.4 million animals. The emergence of resistance to chemical anthelmintics has posed significant challenges to sheep farming [2], as parasitic infections continue to cause considerable economic and productivity losses [3, 4]. Among the primary obstacles in Brazilian ovine production, gastrointestinal parasitism remains one of the most pressing issues, contributing substantially to financial losses for producers [5]. Infected sheep may suffer from weight loss, recurrent diarrhea, severe anemia, reduced wool and milk output, diminished reproductive performance, and in severe cases, mortality [6]. The predominant gastrointestinal endoparasites affecting ruminants include *Haemonchus contortus*, *Trichostrongylus colubriformis*, *Oesophagostomum columbianum*, and *Strongyloides papillosus* [7], which are widely recognized as the principal etiological agents of parasitic infections in sheep [8]. Consequently, the adoption of effective control strategies is imperative [9].

Synthetic anthelmintics are commonly employed to control gastrointestinal nematodes (GINs) [10]. Nevertheless, their indiscriminate application and lack of appropriate dosing have diminished therapeutic efficacy, thereby facilitating the development of parasite resistance, increasing mortality rates, and elevating treatment costs [4, 11]. Furthermore, improper packaging and handling practices may lead to residual contamination [12], as misadministration often results in the excretion of drug residues by treated animals [13]. Of increasing concern is the contamination of non-target organisms by improperly used anthelmintics. This has been substantiated by studies involving algae, annelids, coleopterans, and aquatic bioindicators such as *Daphnia pulex* and *Danio rerio*, which have demonstrated the environmental presence of these chemical residues [14–16].

Given the limitations of conventional anthelmintics, phytotherapeutic alternatives have emerged as promising agents for managing parasitic diseases in both humans and animals [17]. These plant-derived products exhibit documented biological activity against fungi [18], bacteria [19], ectoparasites [20], and endoparasites [21]. Moreover, certain botanicals have demonstrated homeopathic efficacy, as noted by Dantas [22].

Previous research by Oliveira *et al*. [20] has demonstrated the capacity of wood vinegar (WV) to suppress tick egg hatching. In addition, WVs derived from eucalyptus wood and aromatic herbs through co-pyrolysis have shown effective antimicrobial properties [19]. As a byproduct of charcoal production [23], WV primarily comprises water (90%) and a variety of organic substances (10%), including alcohols, ketones, esters, phenols, hydrocarbons, and other minor constituents [20–24].

Despite the widespread use of synthetic anthelmintics in controlling GINs in small ruminants, their efficacy is increasingly compromised by the emergence of drug-resistant parasite strains, poor dosage practices, and environmental contamination. Although plant-based compounds have demonstrated promising anthelmintic activities, including essential oils and extracts from *Eucalyptus* and *Origanum* species, their application remains largely limited to larvicidal and adulticidal effects. The potential of WV, a pyrolysis-derived bioproduct, as an ovicidal agent remains underexplored. Furthermore, there is a lack of empirical data assessing the synergistic effects of co-pyrolyzed aromatic herbs with hardwoods in enhancing the ovicidal activity of WV. To date, no published studies have examined the impact of WV obtained from co-pyrolysis of *Eucalyptus* wood and *Origanum majorana* on the hatchability of GIN eggs in sheep under controlled *in vitro* conditions nor have they characterized the chemical constituents responsible for such activity through gas chromatography-mass spectrometry (GC/MS).

This study aimed to evaluate the *in vitro* ovicidal efficacy of eucalyptus WV (clone I144) and a modified formulation obtained by co-pyrolyzing eucalyptus wood with *O. majoran*a against the eggs of GINs from sheep. In addition, the chemical composition of both WVs was analyzed using GC/MS to identify potential bioactive compounds. Scanning electron microscopy (SEM) was employed to observe structural alterations in eggs post-treatment, providing mechanistic insights into the antiparasitic effects. By addressing the ovicidal potential of these natural products, the study contributes to the development of sustainable, plant-based alternatives to synthetic anthelmintics in the management of parasitic infections in small ruminants.

## MATERIALS AND METHODS

### Ethical approval

**Figure 1:**
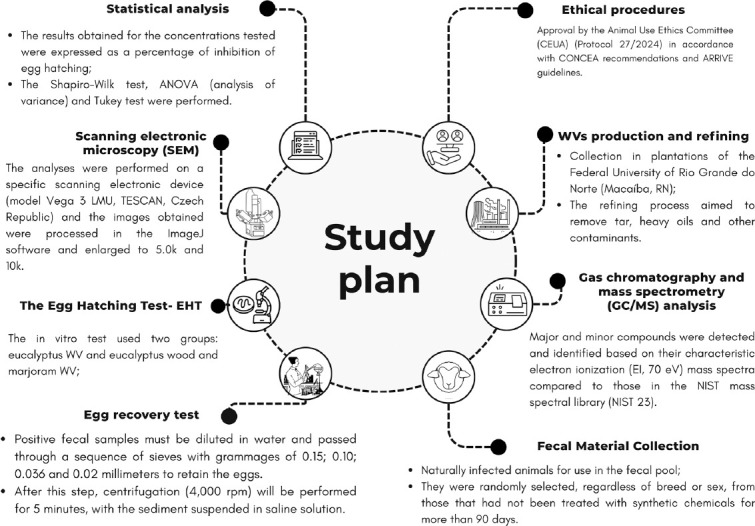
Study plan.

This study was approved under protocol number 27/2024 by the Animal Use Ethics Committee, in accordance with the guidelines established by the National Council for Animal Control and Experimentation and the ARRIVE guidelines. The ethical approval permitted animal handling for sample collection within the municipality of Mossoró, Rio Grande do Norte State, Brazil (coordinates: 05°11′16.8″ S, 37°20′38.4″ W). A schematic of the study design is presented in Figure 1.

### Study period and location

The study was carried out from July 2023 to August 2024 in the municipalities of Mossoró and Natal, located in the State of Rio Grande do Norte, Brazil.

### WV production and refinement

The WV samples were produced by pyrolysis following protocols described by Gama *et al*. [19] and Pimenta *et al*. [25]. Two types of WV were obtained: One derived solely from eucalyptus wood (hybrid of *Eucalyptus urophylla × Eucalyptus grandis*, clone I144) and another generated through co-pyrolysis with *O. majorana* (marjoram). (A) Eucalyptus wood was harvested from plantations at the Escola Agrícola de Jundiaí, Universidade Federal do Rio Grande do Norte, Macaíba, Brazil. (B) The wood was sectioned into 3 cm discs, quartered, and oven-dried for 48 h before carbonization (10 replicates). (C) The resulting raw pyrolysis liquids were combined into a composite sample and stored at 6°C, then subjected to ultrafiltration and sequential vacuum distillation to obtain refined WV. (D) The final product was stored in autoclave-sterilized amber glass vials at 120°C. Refinement aimed to eliminate tars, heavy oils, and contaminants as outlined by Pimenta *et al*. [26].

### GC/MS analysis

(A) From 100 mL WV batches, 5 mL aliquots were collected. Each was alkalized with 1.5 mL of concentrated ammonium hydroxide (Caledon, UN 2672, Canada) to reach pH ~7.0. (B) Liquid-liquid extraction was performed using three successive 3 mL volumes of high-performance liquid chromatography-grade ethyl acetate (Merck, São Paulo, Brazil). (C) 1 mL of the organic extract was transferred to GC vials for immediate analysis using a Shimadzu QP 2010 single quadrupole GC/MS system. (D) Chromatographic separation employed a DB-Wax 52 CB column (30 m × 0.25 mm, 0.25 μm film thickness; Agilent), with injector temperature at 250°C. Injections (1 μL) were made in split mode (1:10). The oven program was 50°C for 2 min, ramped at 2°C/min to 240°C, with a 2-min hold. Helium served as the carrier gas at 1 mL/min flow rate. The total run time was 99 min. (F) Each WV type was injected in triplicate. (G) Chromatograms and spectra were highly reproducible. (H) Compounds were identified by electron ionization (70 eV) spectra, matched against the NIST library (NIST05s.LIB e NIST05.LIB Software, version 2023), with major compounds showing ≥85% spectral similarity and minor ones ≥80%.

### Egg collection, preparation, and egg hatchability testing (EHT)

Sheep used for fecal collection were naturally infected with prevalent gastrointestinal parasites and had not received anthelmintic treatment in the preceding 90 days, as described by Niciura *et al*. [27]. (A) Fecal samples were obtained rectally and analyzed for egg counts per gram (EPG), following Gordon and Whitlock [28] and Chagas [29]. (B) Only samples with EPG ≥2000 were included [29]. (C) Feces were pooled for coproculture and L3-stage larval identification [30]. The remaining feces were processed for egg isolation, with each concentration assessed in five replicates across five independent collections (25 total replicates). (D) Egg isolation followed the method by Hubert and Kerboeuf [31], involving sequential sieving (0.15–0.02 mm) and centrifugation (1800 × *g* for 5 min), followed by resuspension in saline. (F) EHTs were performed as per Coles *et al*. [32] by placing ~100 eggs (100 μL in each well with 400 μL of WV at varying concentrations in sterile 24-well plates (five wells per concentration). (G) Treatments included WVs from eucalyptus alone and eucalyptus + marjoram at 0.3125%, 0.625%, 1.25%, 2.5%, and 5% (g/100 mL). The 5% dose was the minimum lethal concentration. Controls included distilled water (negative) and thiabendazole (32 μL/mL; Sigma- St. Louis, Missouri, USA) in 1% Dimetilsulfóxido (positive). (H) Plates were incubated at 27°C in a biochemical oxygen demand chamber (SOLAB, São Paulo) for 48 h. Lugol’s solution was added, and egg and L1 counts were performed using an inverted microscope.

### SEM

(A) Nematode eggs were fixed in Karnovsky’s solution (0.1 M sodium phosphate buffer, pH 7.4) for 24 h. (B) Post-fixation involved 1% osmium tetroxide in the same buffer for 2 h, followed by buffer rinsing. (C) Samples were sequentially dehydrated in ethanol (7.5%–70%, 10 min each). (D) Fully dehydrated samples were dried at 37°C. (E) Specimens were mounted on stubs, sputter-coated with 35 nm gold-palladium, and imaged using a Vega 3 LMU scanning electron micro-scope (TESCAN, Czech Republic). Images were processed with ImageJ and magnified at 5,000× and 10,000×. Six slides were examined for the control and 5% euca-lyptus WV groups, with an average of 30 eggs per slide.

### Statistical analysis

Egg hatch inhibition (%) was calculated as (number of eggs/[number of eggs + number of L1 larvae]) × 100. Data were expressed as percentage inhibition per concentration. Normality was assessed using the Shapiro–Wilk test (p > 0.05). Differences among means were evaluated using analysis of variance followed by Tukey’s *post hoc* test at a 95% confidence level. All statistical analyses were performed using Statistica version 14.0.1 (TIBCO Software Inc., Palo Alto, CA, USA).

## RESULTS

### WV production and refining

Table 1 presents the carbonization results for eucalyptus wood and its combination with marjoram. The WV yields obtained from the co-pyrolysis of eucalyptus wood with marjoram were comparable to those from the carbonization of eucalyptus wood alone. Including marjoram did not significantly increase the yield of pyrolysis liquids, despite producing a similar gas profile to the control (eucalyptus wood only). This outcome aligns with findings reported in the literature regarding eucalyptus clones [25, 33] and industrial carbonization processes [26]. In both experimental treatments, the raw pyrolysis liquid yields exceeded 40%, which is notable from an industrial perspective, as higher yields of both charcoal and condensable liquids enhance economic viability due to their commercial value. The yields of purified WV following vacuum distillation showed no significant differences between the two treatments. In both cases, the refinement efficiency exceeded 95%, which concurs with the results previously reported by Pimenta *et al*. [26].

**Table 1:** Gravimetric yields determined during carbonization runs.

Material	Gravimetric yield (%)*		

	Charcoal	Liquids	Gases
Eucalyptus wood	34.9	43.1	22.0
Wood + marjoram: The Count of Marjorams.	33.8	41.1	25.1

* Gravimetric yields in charcoal, pyrolysis liquids, and gases were calculated based on the initial weight of bone-dry wood and bone-dry wood + marjoram

Refining is crucial for removing heavy oils and tar pitch, commonly present in WV. Distillation remains the most effective and reliable method for producing high-quality WV products [26, 34]. The distillation process thoroughly eliminates polycyclic aromatic hydrocarbons, volatile organic compounds, and other undesirable contaminants from the WV matrix [26, 35]. Consequently, the biological activity observed in the refined WV can be attributed solely to its active organic constituents.

### GC/MS analysis

Table 2 presents the annotated chemical constituents identified in eucalyptus WV and the formulation obtained by co-pyrolysis with marjoram. Based on GC/MS analysis, the chemical profiles of both products were generally similar, comprising a combined total of 103 compounds, with 42 compounds commonly detected in both types of WV. Nonetheless, notable differences were observed: Some compounds were reduced or absent in one formulation, while new compounds emerged in the marjoram-enriched WV. Specifically, 23 compounds were unique to the eucalyptus-only WV, whereas 38 compounds were exclusive to the eucalyptus WV produced with marjoram. Among these, thymol and eucalyptol – bioactive constituents known for antimicrobial and antiparasitic properties – were detected in the marjoram-associated WV at concentrations of 0.20% and 0.21%, respectively. Phenolic compounds and furfural were prominent in both formulations. However, furfural was the dominant constituent in the eucalyptus-marjoram WV, reaching a peak area of 10.52%. In contrast, phenol was notably abundant in the eucalyptus-only WV, at 9.34%.

**Table 2:** Annotated compounds in eucalyptus wood from WVs and eucalyptus wood + marjoram.

Compound	WV type	

	Eucalyptus wood	WV (Wood + marjoram)
Furfural	8.92	10.52
2-methoxy-phenol	12.35	11.95
Phenol	9.34	*
Borazine, 2,4-dimethyl-	*	4.95
2-Furancarboxaldehyde, 5-methyl-	3.49	3.46
Phenol, 2-methoxy-4-methyl-	5.98	4.07
2-Cyclopenten-1-one	1.87	3.55
2-Cyclopenten-1-one, 2-methyl-	0.95	3.29
Hexanoic acid, 6-bromo-	2.47	*
Acetic acid	2.45	6.19
1-Hydroxy-2-butanone	0.82	2.22
Butanoic acid	*	2.11
2-Cyclopenten-1-one, 3-methyl-	2.36	2.28
2-Cyclopenten-1-one, 2,3-dimethyl-	1.88	1.83
Cyclopentanone	*	1.79
Methyl 2-furoate	*	1.55
Propanoic acid	1.43	1.85
Phenol, 3-methyl-	3.87	1.17
2-Cyclopenten-1-one, 2,3-dimethyl-	*	1.24
Phenol, 4-methyl-	2.58	0.97
2-Butanone, 1-(acetyloxy)-	*	0.92
Phenol, 3,4-dimethyl-	0.8	*
1,2-Cyclopentanedione, 3-methyl-	2.18	0.78
N-Nitrosodimethylamine	0.09	0.8
Phenol, 2-methoxy-3-methyl-	0.16	0.77
2-Cyclopenten-1-one, 3,4-dimethyl-	0.44	0.72
2,3-Pentanedione	0.57	0.73
3-(Diethylamino)-1,2-propanediol	0.11	0.71
Pyridine	1.02	0.69
Phenol, 2,6-dimethoxy-	10.3	0.7
2-Furanmethanol	*	0.64
2-Cyclopenten-1-one, 3-ethyl-	0.55	0.56
2,6-Heptadien-1-ol, 2,4-dimethyl-	0.56	*
3-Cyclohexen-1-ol, 4-methyl-1-(1-methylethyl)-, (R)-	*	0.54
2-Butanone, 1-(acetyloxy)-	0.51	*
2-Cyclopenten-1-one, 2,3,4-trimethyl-	0.10	0.51
2-Butenal, 2-ethyl-	0.48	*
2-Methoxy-5-methylphenol	*	0.47
Furan, 2-(methoxymethyl)-	*	0.46
Phenol, 2,4-dimethyl-	*	0.58
1,2,3-Trimethoxybenzene	0.45	*
Bicyclo[2.2.2]octane, 2-methyl-	0.17	0.43
Phenol, 2,6-dimethyl-	0.08	0.40
3-Ethenyl-3-methylcyclopentanone	*	0.40
Butanoic acid, 2-hydroxymethyl ester	*	0.40
Pentanoic acid, 3-methyl	0.39	0.84
2-Cyclopenten-1-one, 2,3-dimethyl-	*	0.39
2-Cyclopenten-1-one, 3-ethyl-2-hydroxy-	0.20	0.38
Cyclohexene, 1-isopentyl-	*	0.38
(R)-(+)-3-Methylcyclopentanone	*	0.38
Phenol, 2-methoxy-4-methyl-	0.40	*
2-Cyclopenten-1-one, 3-ethyl-2-hydroxy-	0.73	0.36
Pentanoic acid, 4-methyl-	*	0.35
2,3-Dimethoxytoluene	*	0.35
1,3-Hexadiene, 3-ethyl-2-methyl, (Z)-	*	0.34
Cyclopentanone, 2-methyl-	*	0.33
Ethylbenzene	0.43	0.33
Phenol, 3,5-dimethyl-	0.34	0.47
Cycloheptanone, 2-ethyl-	0.31	*
Pentanoic acid	0.38	0.31
2,4-Pentadien-1-ol, 3-propyl-, (2Z)-	0.30	*
Pyrazine, methyl-	*	0.29
Adrenalone	*	0.28
2-Acetyl-5-methyl furan	0.29	0.28
2,5-Hexanedione	0.28	*
Vinyl butyrate	*	0.28
2-Butanol, 1-methoxy-	0.07	*
Cyclohexaneacetic acid, -ethyl-	*	0.28
Phenol, 2-methoxy-4-propyl-	0.21	0.27
1,4-Dioxin, 2,3-dihydro-	0.05	0.26
2-Hepten-3-ol, 4,5-dimethyl-	0.24	*
2-Cyclohexen-1-one	0.07	0.24
Oxalic acid, isobutyl pentyl ester	*	0.24
3-Pentanol	*	0.24
2-Cyclopenten-1-one, 3-methyl-	0.04	*
3-Furaldehyde	*	0.23
2-Propen-1-ol	*	0.25
4-Hepten-3-one, 4-methyl-	*	0.22
Maltol	0.21	*
Furan, 2,5-dihydro-3,4-dimethyl-	*	0.21
2(3H)-Furanone, dihydro-5-methyl-	0.21	*
Pyridine, 2-methyl-	0.07	0.21
Eucalyptol	*	0.21
Phenol, 2-ethyl-	0.09	0.20
2-Cyclopenten-1-one, 3-ethyl-2-hydroxy-	0.57	0.20
2,3-Pentanedione	0.03	0.20
2-Hydroxy-3-propyl-2-cyclopenten-1-one	0.19	*
4-Hexen-3-one, 4,5-dimethyl-	0.31	*
Butyrolactone	0.39	0.19
2-Cyclohexen-1-one, 2-methyl-	*	0.19
3-Heptyne	*	0.19
Benzene, 1,3-dimethyl-	0.19	*
Cyclooctene	*	0.18
2-Hexanol, 2-methyl-	*	0.18
1,2,3-Trimethoxybenzene	0.2	*
Cyclohexane, (1-methylethylidene)-	0.17	*
Thymol	*	0.20
Ethanone, 1-(2-furanyl)-	1.08	3.72
2H-Pyran-3(4H)-one, dihydro-	0.08	0.16
p-Xylene	*	0.16
Pentanoic acid, 3-methyl	*	0.15
2-Cyclopenten-1-one, 3-ethyl-2-hydroxy-	0.15	*
3-Penten-2-ol	0.15	*

In the table, asterisks (*) indicate that the compound was not annotated in the respective WVs. WV=Wood vinegar. Numbers in the table are peak areas (%)

### EHT

**Figure 2:**
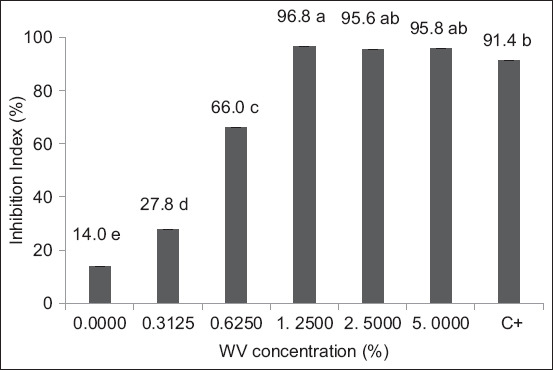
Effect of eucalyptus WV on the hatchability of sheep nematode eggs according to nematode concentration. *Means followed by the same letters are not statistically different by the Tukey test at a 95% probability; 0.0000 concentration = negative control; C+ = Positive control. WV = Wood vinegar.

**Figure 3:**
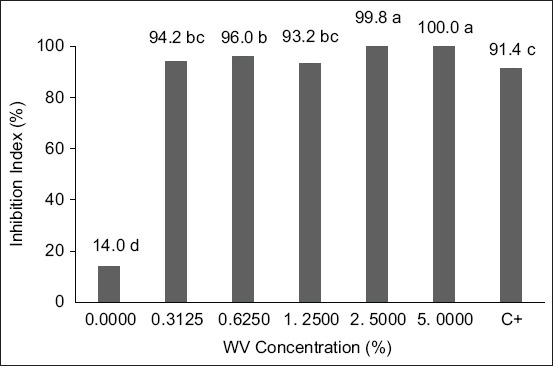
Effects of eucalyptus WV plus marjoram on the hatchability of sheep nematode eggs according to the concentration of eucalyptus. *Means followed by the same letters are not statistically different by the Tukey test at a 95% probability; 0.0000 concentration = negative control; C+ = Positive control. WV = Wood vinegar.

Figure 2 presents the results of egg hatch inhibition in sheep nematodes following exposure to eucalyptus WV. The highest levels of inhibition were observed at the 5% and 2.5% concentrations. In contrast, the WV derived from eucalyptus co-pyrolyzed with marjoram exhibited consistent ovicidal activity across all tested concentrations. However, statistical analysis revealed no significant differences between the highest (5% and 2.5%) and the lowest concentrations tested (Figure 3).

**Figure 4:**
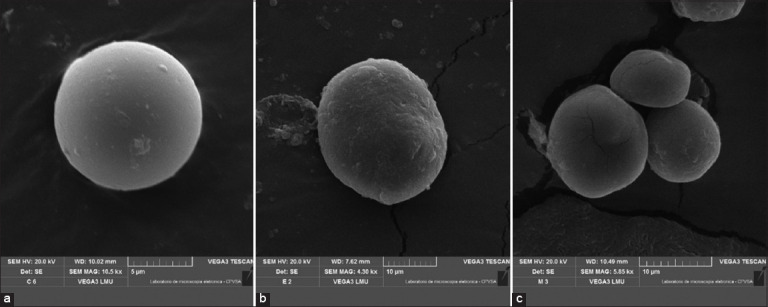
Micrographs obtained by electronic scanning microscopy for nematode eggs subjected to the effect of two types of WV: (a) Negative control, magnification 5 k, (b) Eucalyptus WV, and (c) Eucalyptus WV plus marjoram; magnification 10k—WV=Wood vinegar.

Coproculture analysis confirmed the presence of nematodes from the genera *Haemonchus* spp., *Trichostrongylus* spp., *Oesophagostomum* spp., and *Strongyloides* spp. in all fecal samples used for egg recovery. Figure 4 displays SEM images of eggs from the control group and those treated with 5% WV. This concentration was selected for imaging due to its demonstrated high inhibitory efficacy in both WV treatments. Notably, eggs exposed to WV exhibited marked structural alterations in their membranes compared to the control. While eggs from the control group retained their characteristic elliptical and symmetrical morphology with intact membranes, those treated with WV showed clear morphological deformations, likely attributable to the bioactive effects of the vinegar formulations.

## DISCUSSION

Newcomers to the study of WV often question why the isolated active compounds are not employed directly, considering that WV contains over 200 chemical constituents, of which only approximately 20 have established biological activity. Several authors have addressed this issue, noting that the isolation and purification of individual compounds would be prohibitively expensive and economically unviable for practical applications. Moreover, a key feature of WV lies in the synergistic interaction among its constituents – wherein one compound may enhance the biological efficacy of another. This synergy results in a cumulative effect greater than the sum of individual actions [19, 36, 37]. In addition, WV remains an economically viable option due to its low cost on the international market, further negating the justification for chemical fractionation [26].

Extensive research has demonstrated the potential of WVs as sustainable alternatives to synthetic agents, exhibiting nematocidal, ectoparasiticidal, antiviral, and antimicrobial properties [19, 20, 36, 38–41]. As natural products derived from biomass pyrolysis, WVs represent a renewable and environmentally friendly option. Furthermore, the recovery of WV from wood carbonization smoke contributes to reducing environmental contamination in industrial charcoal production systems [26, 42]. In the present study, eucalyptus WV and the formulation enriched with *O. majorana* showed maximal ovicidal efficacy at 1.25% and 5% concentrations, inhibiting 97% and 100% of egg hatchability, respectively.

Supporting these findings, other studies involving compounds from the *Eucalyptus* genus have reported high anthelmintic efficacy. For instance, nanoemulsions of *Eucalyptus globulus* essential oil at 5% concentration achieved 84% inhibition of egg hatching and 98% inhibition of larval development [43]. Similarly, Macedo *et al*. [44] reported 99.3% inhibition of egg hatchability and 98.7% inhibition of larvae using *E. globulus* essential oil. These results provide a comparative framework for the present findings. In this study, the eucalyptus-marjoram WV also inhibited 97% and 100% of egg hatchability at 1.25% and 5%, respectively. Although the concentration of eucalyptol in the experimental formulation reached only 0.21%, and thymol 0.20%, their presence likely contributed to the enhanced ovicidal activity through synergistic interactions with other WV constituents.

These bioactive compounds – thymol and eucalyptol – were present in lower concentrations in the eucalyptus-marjoram WV. However, their synergistic interaction has been widely studied for its potential to enhance antimicrobial and antiparasitic effects [45]. Thymol destabilizes microbial cell membranes, while eucalyptol facilitates compound penetration and efficacy within biological systems [46]. This interaction may increase cytotoxicity and membrane permeability, thereby enhancing therapeutic efficacy in antiparasitic applications [47]. Essential oils from *O. majorana* have shown documented biological activities, including antimicrobial effects against *Acinetobacter baumannii* [48]. Furthermore, Abidi *et al*. [49] demonstrated that *O. majorana* essential oil reduced egg counts by 76.3% and adult worm counts by 74% in treated animals after 7 days.

Furfural was one of the most abundant and significant compounds in both WV types evaluated in this study. Its presence, combined with phenolic compounds and marjoram-derived constituents, appears to confer robust biological activity. As a byproduct of biomass carbonization, furfural has demonstrated strong inhibitory effects on parasite development [50]. Fabiyi [51] reported that furfural effectively disrupted the life cycle of plant-parasitic nematodes, causing cytotoxic damage and reducing their survival and pathogenicity. Similarly, Ortu *et al*. [52] identified a marked inhibitory effect of furfural on GINs of sheep, particularly in the third larval stage (L3). This effect is attributed to furfural’s capacity to interfere with essential cellular processes, such as mitochondrial function and protein synthesis. The inhibitory impact observed on egg hatchability in the current study is thus consistent with previously reported bioactivity.

Phenolic compounds, abundant in both WV formulations, also merit consideration. These organic compounds are recognized for their wide-ranging biological activities, including potent anthelmintic effects [53–58]. Due to their structural properties, phenolics can impair parasite viability. Previous studies by Costa *et al*. [59] and Vieira *et al*. [60] demonstrated that phenolic compounds, either alone or in combination with tannins, exert synergistic antiparasitic effects.

The SEM analysis revealed intact, elliptical eggs with smooth shells in the negative control group, consistent with typical morphology observed under natural conditions. These protective external layers shield the larvae from mechanical and chemical insults [61]. Silveira *et al*. [62] observed that nematodes possess robust outer membranes functioning as defensive barriers. However, eggs treated with WV displayed pronounced structural changes. Bortoluzzi *et al*. [63] reported similar findings, showing membrane disruption in nematode eggs exposed to bioactive plant extracts. Yoshihara *et al*. [64] also observed decreased membrane thickness and increased fragility following exposure to antiparasitic agents. Comparable effects were demonstrated with bamboo (*Bambusa vulgaris*) WV, which altered the permeability and viability of *Meloidogyne incognita* eggs in lettuce [65]. As described by Pereira *et al*. [66], both condensable and non-condensable fractions of WV disrupt endoparasite egg membranes, increasing permeability and compromising larval viability. These findings collectively corroborate the present study’s results, confirming that WV formulations significantly reduce the hatchability of nematode eggs.

## CONCLUSION

This study demonstrated the potent ovicidal activity of eucalyptus WV, particularly when enhanced through co-pyrolysis with *O. majorana*, against GIN eggs from sheep. Both WV formulations significantly inhibited egg hatchability *in vitro*, with eucalyptus WV achieving 97% inhibition at a 1.25% concentration and the eucalyptus–marjoram WV attaining 100% inhibition at 5%. GC/MS revealed the presence of biologically active compounds such as phenolics, furfural, thymol, and eucalyptol, which are likely responsible for the observed anthelmintic effects. SEM confirmed that WV exposure induced substantial morphological damage to nematode egg membranes, reinforcing its mechanism of action.

The study’s main strength lies in its comprehensive experimental design, combining chemical characterization with biological efficacy and structural validation. Furthermore, it provides novel evidence supporting the synergistic potential of marjoram-derived compounds when co-pyrolyzed with eucalyptus wood – a sustainable strategy for enhancing WV’s antiparasitic efficacy. The use of naturally infected animals and a robust sample replication framework enhances the ecological validity of the findings.

Nevertheless, the study is subject to certain limitations. The ovicidal effects were assessed under controlled *in vitro* conditions, which may not fully reflect the complexity of *in vivo* gastrointestinal environments. In addition, the chemical interactions and precise mechanisms of action among the WV constituents warrant further elucidation through molecular or proteomic approaches. The study also did not evaluate potential toxicity or long-term effects of WV formulations on animal health and soil ecosystems.

Future investigations should prioritize *in vivo* trials to assess efficacy, safety, and dosage optimization in livestock systems. In addition, studies exploring the mode of action of key compounds, particularly furfural, thymol, and eucalyptol, through omics-based techniques, could elucidate synergistic pathways. Integrating WV formulations into integrated parasite management programs could offer a sustainable, low-cost alternative to synthetic anthelmintics, particularly in regions facing escalating drug resistance and environmental concerns.

## AUTHORS’ CONTRIBUTIONS

YTRM: Conducted all experiments, collected and tabulated experimental data, interpreted experimental data, wrote the manuscript, and prepared the final Portuguese version. ASP: Overall coordination of research activities and fundraising, interpretation of experimental and statistical data and translated the manuscript from Portuguese to English. LCDM: Performed the carbonization runs, prepared and refined the pyroligneous acids, and allocated supplies and reagents. MFO: Coordinated and assisted in the SEM analyses. AKAF and AMSP: Assisted in the experiments and collected fecal samples. ACDSB: Coordinated the experiments, allocated supplies and reagents, interpreted experimental data, and reviewed the draft and final versions of the manuscript. TVCM: Performed the GC/MS analyses, acquired the chromatograms and mass spectra, and tabulated the experimental data. MF: Coordinated the GC/MS analysis, interpreted the chromatograms and mass spectra, prepared the GC data table, interpreted the experimental data, and reviewed the final version of the manuscript. RRM: Performed the statistical analyses of the experimental data. All authors have read and approved the final manuscript.
